# Antiviral resistance during pandemic influenza: implications for stockpiling and drug use

**DOI:** 10.1186/1471-2334-9-8

**Published:** 2009-01-22

**Authors:** Julien Arino, Christopher S Bowman, Seyed M Moghadas

**Affiliations:** 1 Department of Mathematics, University of Manitoba, Winnipeg, Manitoba R3T 2N2, Canada; 2 Institute for Biodiagnostics, National Research Council Canada, Winnipeg, Manitoba R3B 1Y6, Canada; 3 Department of Electrical and Computer Engineering, University of Manitoba, Winnipeg, Manitoba R3T 5V6, Canada; 4 Department of Mathematics and Statistics, University of Winnipeg, Winnipeg, Manitoba R3B 2E9, Canada; 5 Department of Statistics, University of Manitoba, Winnipeg, Manitoba R3T 2N2, Canada

## Abstract

**Background:**

The anticipated extent of antiviral use during an influenza pandemic can have adverse consequences for the development of drug resistance and rationing of limited stockpiles. The strategic use of drugs is therefore a major public health concern in planning for effective pandemic responses.

**Methods:**

We employed a mathematical model that includes both sensitive and resistant strains of a virus with pandemic potential, and applies antiviral drugs for treatment of clinical infections. Using estimated parameters in the published literature, the model was simulated for various sizes of stockpiles to evaluate the outcome of different antiviral strategies.

**Results:**

We demonstrated that the emergence of highly transmissible resistant strains has no significant impact on the use of available stockpiles if treatment is maintained at low levels or the reproduction number of the sensitive strain is sufficiently high. However, moderate to high treatment levels can result in a more rapid depletion of stockpiles, leading to run-out, by promoting wide-spread drug resistance. We applied an antiviral strategy that delays the onset of aggressive treatment for a certain amount of time after the onset of the outbreak. Our results show that if high treatment levels are enforced too early during the outbreak, a second wave of infections can potentially occur with a substantially larger magnitude. However, a timely implementation of wide-scale treatment can prevent resistance spread in the population, and minimize the final size of the pandemic.

**Conclusion:**

Our results reveal that conservative treatment levels during the early stages of the outbreak, followed by a timely increase in the scale of drug-use, will offer an effective strategy to manage drug resistance in the population and avoid run-out. For a 1918-like strain, the findings suggest that pandemic plans should consider stockpiling antiviral drugs to cover at least 20% of the population.

## Background

Future outbreaks of emerging infectious pathogens are virtually certain to occur, and pandemic influenza is one that seemingly poses a significant threat to human populations. While the characteristics of the next pandemic strain remain unknown, the virulence of the currently circulating avian influenza A virus H5N1 is of great concern [1,2]. Given uncertainties regarding the timing, origin, and virulence of future pandemic strains, as well as the possibility of an unprecedented spread of the deadly H5N1 virus in humans, planning strategies for an effective response has become the top priority of global public health efforts [3-7].

Pandemic preparedness measures encompass disease surveillance, case identification and treatment, prevention of community-wide spread of disease, maintenance of essential services, and research and evaluation [8]. Specific approaches to influenza infection control include the use of pharmaceutical products (such as vaccines and antiviral drugs), and non-pharmaceutical measures (such as personal protective equipment and social distancing). Although vaccination remains the most effective strategy for reducing the risk of infection and subsequent complications [9], an effective vaccine may not be available for several months following the declaration of a pandemic. This highlights the importance of antiviral drugs as the primary tool for prevention and treatment of infection [10], especially in light of the insufficient impact that non-pharmaceutical measures may have on disease mitigation [11].

Considering that there may be insufficient supply of drugs, limited production capacity, and a surge in demand for antiviral therapy with the progression of a pandemic, the use of antivirals for treatment will likely take precedence over their preventive (prophylactic) use. The primary goal in treatment of influenza infection is to relieve symptoms and limit the severity of infection by inhibiting virus replication. This will in turn contribute to the containment of disease spread in the population as a result of reduced viral transmission. It is therefore imperative to formulate antiviral policies that are most likely to optimize the health of the greatest number of individuals in the face of an influenza pandemic.

Although antiviral treatment appears to be crucial in any pandemic response, the emergence of drug-resistance will impose significant threats to the effectiveness of drugs [12-17], and possibly wasteful depletion of stockpiles without achieving the desired mitigation impact. Previous modelling studies suggest that, in the ideal situation where adequate supply of antiviral drugs is secured, conservative treatment levels at the early stages of the outbreak, followed by a timely increase in the scale of drug-use, would preserve the potential for minimizing the final size of a pandemic while preventing large outbreaks of drug-resistant infections [18,19]. In this study, we further investigate the merits of application of antiviral treatment under the scenario in which the supply of drugs may be limited and run-out is possible. By employing a mathematical model, we show the relationship between the drug stockpile and treatment level (the fraction of clinical infections being treated) for a range of reproduction numbers estimated for the past three pandemics [20]. We discuss the influence of emergence of antiviral resistance for the use of drugs and demonstrate possible scenarios of disease outbreaks, including a second wave of infections in a single outbreak. Our findings extend a previous work [21], in which emergence and transmission of resistance are neglected. We also evaluate the impact of an adaptive antiviral strategy on disease mitigation [18,19], where aggressive treatment of clinical infections is delayed for a certain amount of time after the onset of the outbreak. Finally, we discuss model predictions and their implications for stockpiling and drug use in pandemic planning.

## Methods

**Figure 1:**
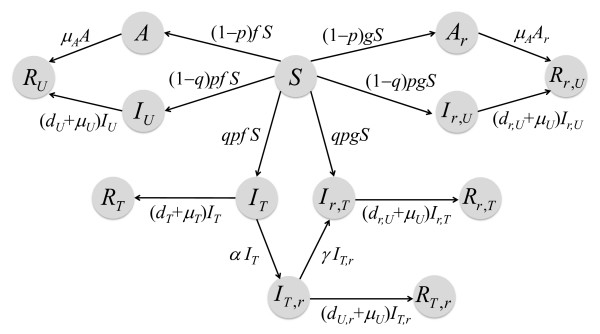
**Model structure**. Model diagram for the progression of disease and development of drug-resistance during treatment of infected individuals.

We followed a deterministic modelling approach and divided a population of size *S*_0 _(that is entirely susceptible to the emergent strain) into classes of susceptible (*S*), asymptomatically infected (*A*), symptomatically infected (*I*), and removed (recovered/dead) individuals (*R*). Infected individuals may undergo an asymptomatic (subclinical) infection for the entire course of disease, without being diagnosed, and therefore are not treated. A fraction of those who develop symptomatic (clinical) disease is treated; this is referred to as the treatment level. We considered three strains of the pathogen, namely: drug-sensitive, drug-resistant with low transmission fitness (LTF), and drug-resistant with high transmission fitness (HTF). The latter initially evolves from further replication and (compensatory) mutations of resistant strains with LTF [22]. It is assumed that individuals hosting resistance with LTF make no contribution to the spread of resistant viruses [23-25]. In the model presented here, the number of resistant infections increases through (i) development of drug-resistance during treatment of sensitive infections, and (ii) direct transmission of resistant strains with HTF. We assumed that the treatment has no effect in reducing the level of infectiousness in resistant infections [16,26,27]. Furthermore, it is assumed that infection caused by sensitive or resistant strains results in generation of immunity against all pathogen strains upon recovery. The transitions between classes of individuals are schematically represented in Figure 1 [see additional file 1].

### Reproduction numbers

In order to evaluate the effectiveness of various antiviral strategies, we calculated the control reproduction number as $R_{c}=\max\{R_{c}^{s},R_{0}^{r}\}$ [see additional file 1], where $R_{c}^{s}$ represents the effective reproduction number of the sensitive strain when treatment is applied, and $R_{0}^{r}$ is the reproduction number of the resistant strain with HTF for which treatment is ineffective. The expressions for these quantities are given by

(1) $$R_{c}^{s}=\beta S_{0}\left(\frac{(1-p)\delta_{A}}{\mu_{A}}+\frac{p(1-q)}{d_{U}+\mu_{U}}+\frac{pq\delta_{T}}{d_{T}+\mu_{T}+\alpha }\right),$$

(2) $$R_{0}^{r}=\delta_{r}\beta S_{0}\left(\frac{(1-p)\delta_{A}}{\mu_{A}}+\frac{p}{d_{r,U}+\mu_{U}}\right),$$

where *β *is the baseline transmission rate of the sensitive strain; and description of other parameters with their estimates are given in Table 1. In the absence of treatment (*q *= 0), *R*_*c *_reduces to the basic reproduction number of the sensitive strain given by

**Table 1:** Model parameters and estimations

parameter	description	baseline value	references
1/*μ*_*A*_	mean infectious period of asymptomatic infection	4.1 days	[3,6,7]
1/*μ*_*U*_	mean infectious period of untreated symptomatic infection	4.1 days	[3,6,7]
1/*μ*_*T*_	mean infectious period of treated symptomatic infection	4.1 days	[3,6,7]
*d*_*U*_	death rate of untreated symptomatic infection	0.002 day^-1^	[12,19]
*d*_*T*_	death rate of treated symptomatic infection	0.001 day^-1^	[12,19]
*d*_*U*,*r*_	death rate of resistant infection (low fitness)	0.0002 day^-1^	[12,19]
*d*_*r*,*U*_	death rate of resistant infection (high fitness)	0.0008 day^-1^	[12,19]
*δ *_*A*_	relative transmissibility of asymptomatic infection	0.142	[6,7,19]
*δ *_*T*_	relative transmissibility of treated symptomatic infection	0.4	[3,12,19]
*δ *_*r*_	relative transmissibility of resistant strain (high fitness)	0.9	[19,27]
*p*	probability of developing clinical symptoms	0.67	[6,7]
*α*	rate of emergence of de novo resistance	0.018 day^-1^	[19,25]
*γ*	rate of conversion between resistant mutants	0.0036 day^-1^	[19,25]
*q*	treatment level	variable	

Description of the model parameters with their values obtained from the published literature.

(3) $$R_{0}=\beta S_{0}\left(\frac{(1-p)\delta_{A}}{\mu_{A}}+\frac{p}{d_{U}+\mu_{U}}\right).$$

In single-strain epidemic models, these reproduction numbers can be used to determine the final size of the outbreak [28]. However, the final size relation for multi-strain models, such as the one considered in this study, may be difficult to obtain. If only resistant strains with LTF are present, then the final size of the pandemic can be expressed in terms of the control reproduction number of the sensitive strain, $R_{c}^{s}$ [see additional file 1].

## Results

We considered various scenarios of disease outbreak in the presence of antiviral treatment, when the reproduction number of the sensitive strain (*R*_0_), the treatment level of clinical cases, and the size of drug stockpile vary in their respective ranges. Due to the unknown transmissibility of the pandemic strain, we used reproduction numbers estimated for pandemics of the last century, ranging from 1.5 to 2.5 [20,29]. We simulated the model by introducing a single case infected with the sensitive strain into a susceptible population of size *S*_0_. The rate of de novo resistance (*α*) that generates mutants with LTF is reported to range from 0.018 to 0.072 day^-1 ^[19,25], and we assumed a baseline value of *α *= 0.0018 day^-1^, which results in the emergence of drug-resistance in approximately 6.8% of treated patients in our model. The rate at which treated individuals (hosting resistant viruses with LTF) develop resistance with HTF (90% relative to that of the sensitive strain) is assumed to be 5-fold smaller, taking the baseline value of *γ *= 0.0036 day^-1 ^[19]. These rates contribute to an overall 0.1% incidence of resistance with HTF (without considering direct transmission) in our model. Other parameter values used in simulations are given in Table 1.

### Adequate supply

**Figure 2:**
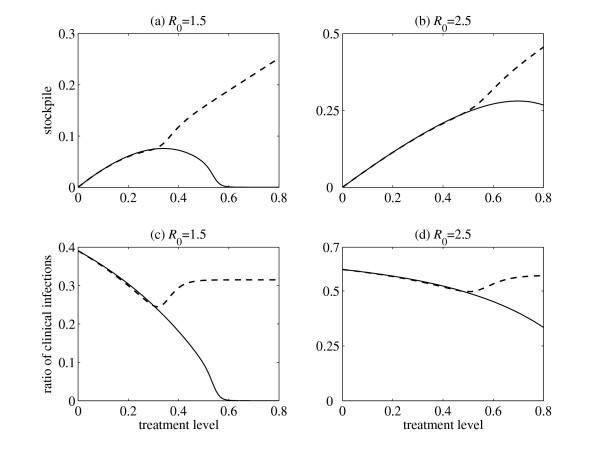
**Required antiviral stockpile and ratio of the total clinical infections**. Required stockpile of antiviral drugs (relative to *S*_0_) as a function of the treatment level for (a) *R*_0 _= 1.5; and (b) *R*_0 _= 2.5. Ratio of the total number of clinical infections to *S*_0 _as a function of the treatment level for: (c) *R*_0 _= 1.5; and (d) *R*_0 _= 2.5. Solid curves correspond to the case where resistance is absent, and dashed curves represent the scenario in which resistant viruses with HTF are present.

We simulated the model by considering a constant treatment level throughout the outbreak. Assuming *R*_0 _= 1.5 ($R_{0}^{r}$ = 1.35), Figure 2a shows the required amount of antiviral courses (relative to the initial size of the population *S*_0_) as a function of the treatment level of clinical infections. As is evident, in the absence of antiviral resistance (*α *= 0), the required stockpile increases as the treatment rises to moderate levels, and decreases for higher treatment levels due to a significant reduction in the spread of the sensitive infection and possible containment of the disease in the population (Figure 2c, solid curve); this is consistent with the results of a previous work [21]. For the scenario considered here, the reproduction number $R_{c}^{s}$ = 1.5 (without treatment) reduces to 1.33 and 1.15 at (low) 20% and (moderate) 40% treatment levels, respectively; and falls below one ($R_{c}^{s}$ = 0.97) as treatment level further increases to 60%, which results in disease containment. However, in the presence of resistance with HTF, the required stockpile will substantially increase for higher treatment levels (Figure 2a, dashed curve), since drug-resistance spreads widely in the population (Figure 2c, dashed curve), resulting in a large number of resistant clinical cases that may receive treatment. This indicates that the supply of antiviral drugs is rapidly depleted, and run-out is more likely to occur should transmissible resistance emerge during the outbreak. Further simulations confirm that these results remain valid for higher values of *R*_0_, as shown in Figure 2b, d for a particular value *R*_0 _= 2.5($R_{0}^{r}$ = 2.25). Figures 2c–d show the corresponding scenarios for the ratio of the total number of clinical cases as a function of the treatment level. Dashed curves show that, if treatment levels are maintained constant, there is an optimal level for minimizing the final size of the epidemic, above which outbreaks with larger magnitudes can occur. The increase in the total number of clinical infections for high treatment levels is consistent with previous observations [19,27], as a direct consequence of emergence of resistant mutants with HTF. We tested the robustness of these results by performing a sensitivity analysis over a wide range of key parameters, including the basic reproduction number, the rate of de novo resistance emergence, the rate of conversion between resistant mutants, the probability of developing clinical disease, and the relative transmissibility of the resistant strain with HTF [see additional file 1]. This analysis demonstrates that (i) intermediate levels of treatment (corresponding to the optimal treatment levels for various samples of parameters) lead to the minimum in the total number of infections; and (ii) a substantially larger stockpile is required when treatment exceeds the optimal level in a constant treatment strategy.

**Figure 3:**
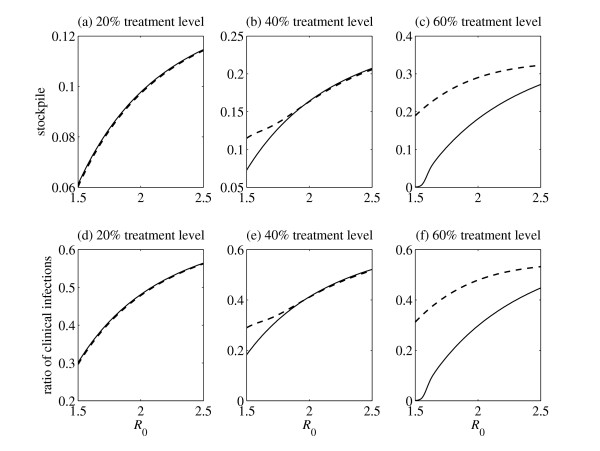
**Unlimited supply of antiviral drugs**. Required stockpile of antiviral drugs (relative to *S*_0_) as a function of *R*_0 _with a constant treatment level of: (a) 20%; (b) 40%; and (c) 60%. Ratio of the total number of clinical infections to *S*_0 _as a function of *R*_0 _with a constant treatment level of: (d) 20%; (e) 40%; and (f) 60%. Solid curves correspond to the case where resistance is absent, and dashed curves represent the scenario in which resistant viruses with HTF are present.

To explore the effect of transmissibility of the pandemic strain on the use of antiviral drugs, we simulated the model for the required antiviral courses (relative to *S*_0_) as a function of *R*_0_, when a constant treatment strategy is implemented [19,27]. Figures 3a–c show the size of stockpile required for 20%, 40%, and 60% treatment levels, when resistance is absent (*α *= 0, solid curves) and present (dashed curves). Clearly, a larger stockpile is needed for higher *R*_0_, especially when resistant mutants with HTF spread in the population. For a low treatment level (20%), the profiles of antiviral courses are virtually identical in both scenarios, as the prevalence of resistance is negligible. For a moderate treatment level (40%), the resistant strain with HTF will gain a competitive advantage for low values of *R*_0_, which requires a significantly larger stockpile compared to the scenario in which resistance is absent (Figure 3b). However, as *R*_0 _increases, the selective advantage of the resistant strain is largely overturned by the high transmissibility of the sensitive strain, and therefore comparable levels of stockpile are required. When treatment is maintained at a higher level (60%), the spread of the sensitive strain is substantially reduced, thereby providing an opportunity for the resistant strain with HTF to out-compete the sensitive strain over available susceptible hosts. Figure 3c shows that, in this case, a significantly larger stockpile is required, which will be largely dispensed for treatment of resistant infections for which it has no effect. The corresponding scenarios for the ratio of the total number of clinical infections as a function of *R*_0 _are illustrated in Figures 3d–f, which corroborate previous findings regarding the wide-spread of antiviral resistance with high treatment levels [18,19,27].

### Inadequate supply

**Figure 4:**
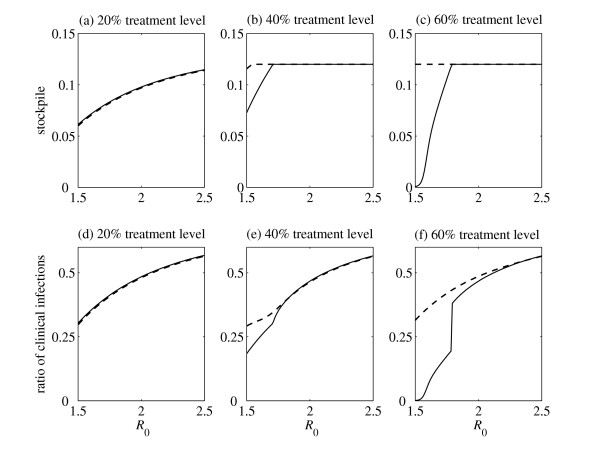
**Limited supply of antiviral drugs**. Antiviral use of an initial 12% stockpile (relative to *S*_0_) as a function of *R*_0 _with a constant treatment level of: (a) 20%; (b) 40%; and (c) 60%. Ratio of the total number of clinical infections to *S*_0 _as a function of *R*_0 _with a constant treatment level of: (d) 20%; (e) 40%; and (f) 60%. Solid curves correspond to the case where resistance is absent, and dashed curves represent the scenario in which resistant viruses with HTF are present.

Assuming that the supply of antiviral courses suffices to treat 12% of the population, we simulated the model for the range of *R*_0 _between 1.5 and 2.5, with different treatment levels. The initial drug supply (12%) amounts to approximately 50% of the targeted size of stockpiles in several national pandemic plans [5,30,31]. In our simulations, we assumed that *q *= 0 when the initial drug supply is entirely dispensed during the outbreak. For a low treatment level (20%), although the disease cannot be contained, drug resistance is unlikely to invade the population and the initial supply would suffice for the duration of the outbreak (Figure 4a). As treatment increases to moderate levels, the supply of drugs is more rapidly depleted and fully dispensed for high values of *R*_0_. Figure 4b shows this scenario for a 40% treatment level, with the occurrence of run-out for smaller *R*_0 _when resistant mutants with HTF are present (dashed curve). We observed similar results for higher treatment levels with run-out for the entire range of *R*_0 _in the presence of transmissible resistance, due to a large number of clinical resistant infections. However, disease containment may be achieved for low values of *R*_0 _when resistance is absent, as illustrated in Figure 4c (solid curve) for a 60% treatment level. These simulations indicate that the emergence of resistance has very little impact on the use of antiviral drugs if treatment is maintained at low levels or the basic reproduction number is sufficiently high. The corresponding ratios of the total clinical infections to *S*_0 _are shown in Figures 4d–f, which highlight the impact of drug resistance on antiviral use with limited stockpile.

### Adaptive treatment strategy

Since emergence of resistant mutants with HTF can potentially result in a rapid depletion of drug stockpiles, management of drug resistance in the population is crucial for the success of any antiviral strategy, in particular when supplies are limited. A recent evaluation of antiviral strategies suggests that, if a pandemic virus is not contained at the source, delaying aggressive treatment can substantially reduce the likelihood of emergence and population-wide spread of resistance [19]. Not only can this adaptive strategy prevent large outbreaks of drug-resistant infections, but it can also minimize the overall pandemic burden if followed by a timely increase in the scale of drug-use.

**Figure 5:**
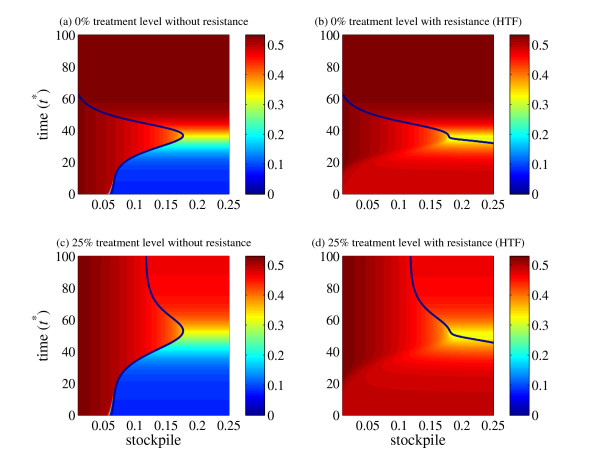
**Final size of infections with adaptive treatment strategy**. The effect of changing treatment level during the outbreak on the total number of clinical infections caused by all strains, with various sizes of stockpile and *R*_0 _= 2. Simulations were seeded with an initial treatment level of: (a) 0% without resistance; (b) 0% with resistance; (c) 25% without resistance; (d) 25% with resistance, and then changed to 80% at the time displayed on the vertical axis (corresponding to the time-course of the outbreak). The color bars illustrate the ratio of the total number of clinical infection to *S*_0 _due to all strains. Run-out occurs in the regions consisting of the origin and delimited by the solid curves.

Here we investigate the effectiveness of an adaptive antiviral treatment as a function of drug stockpile, when the initial treatment level changes at time t* during the outbreak. Assuming *R*_0 _= 2 ($R_{0}^{r}$ = 1.8), Figure 5a shows the ratio of the total number of clinical infections to *S*_0 _in the absence of transmissible resistance, when the initial treatment level 0% increases to 80% at time t* displayed on the vertical axis. In this case, an earlier increase in the treatment level results in a lower number of clinical cases. The region for run-out is delimited by the solid curve, contains the origin, and shrinks as the stockpile increases. In the presence of resistant mutants with HTF, the region for run-out extends to larger stockpiles (Figure 5b, below the solid curve). However, the delay in start of intensive treatment becomes crucial to manage resistance and reduce the total number of infections, particularly when adequate supply of drugs is secured. We observed similar results for an initial 25% treatment level with an extended region for run-out due to higher scale of drug-use (Figures 5c–d). While highlighting the importance of management of drug-resistance in the population, these simulations indicate that for small stockpiles, adaptive antiviral strategy has no significant benefits over a constant treatment policy.

**Figure 6:**
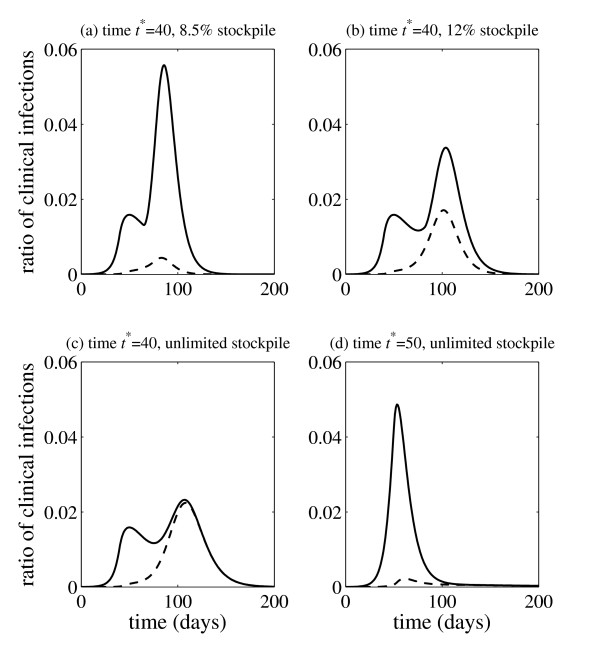
**Time-courses of the outbreak with adaptive treatment strategy**. The effect of changing treatment level during the outbreak on the time course of clinical infections with *R*_0 _= 2. Simulations were seeded with an initial treatment level 25%, and then changed to 80% at: (a) day 40 with stockpile of size 8.5% (relative to *S*_0_); (b) day 40 with stockpile of size 12% (relative to *S*_0_); (c) day 40 with adequate drug supply; (d) day 50 with adequate drug supply. Solid curves show the time courses for the ratio of the clinical infections to *S*_0 _caused by all strains, and dashed curves represent those of only resistant infections.

In order to evaluate the effect of t* and the size of stockpile on disease mitigation, we further simulated the model for the time course of infections with an initial 25% treatment level. Figure 6a shows the profiles of the clinical infections caused by all strains (solid curve) and resistant infections (dashed curve) with a stockpile of size 8.5% (relative to *S*_0_), when treatment increases to 80% at day 40 after the onset of outbreak. As is evident, run-out appears before resistance can widely spread, and a second wave of infections occurs due to a large number of sensitive infections. An increase in the drug supply, and therefore antiviral use, leads to the further reduction in the spread of the sensitive infection, and the effect of resistance becomes more pronounced in the occurrence of the subsequent outbreak, as depicted in Figure 6b for 12% stockpile. We also observed this phenomenon for adequate supply of drugs (Figure 6c); however, the subsequent wave of infections is largely caused by wide-spread drug-resistance. Compared with simulations illustrated in Figure 5d, it can be seen that although the stockpile is not confined, the early onset of aggressive treatment may result in a large resistant outbreak. With adequate quantities of antiviral courses and t* = 50 as the optimal time for raising the treatment level to 80% (Figure 5d), both emergence of resistance and the occurrence of a second wave of infections are prevented, while the final size of the pandemic is also minimized (Figure 6d). We also performed a sensitivity analysis to evaluate the effect of parameters variation on the optimal time t* at which treatment level changes as a function of the reproduction number, in order to minimize the final size of the pandemic [see additional file 1]. Whether the stockpile is limited or not, the results show qualitatively similar patterns, and demonstrate that aggressive treatment should be implemented with shorter delay as the basic reproduction number increases. However, the effect of initial treatment level becomes much more pronounced on the optimal time t*, with significantly longer delay for higher initial treatment levels as the reproduction number decreases.

## Discussion

As nations prepare to confront the next influenza pandemic, disease mitigation strategies are being carefully gauged to project the effectiveness of preventive, therapeutic, and social distancing measures. Published modelling studies suggest that the pandemic can be contained at the source if early treatment of diagnosed cases is combined with targeted blanket prophylaxis and social distancing measures [3,7]. Significant assumptions are embedded in the core of such models, most of which are unlikely to be fulfilled in a real world environment, and therefore containment failure should be anticipated when devising effective preparedness countermeasures.

While application of antiviral drugs has been rationalized as the first-line defence against a pandemic, public health authorities are concerned with the strategic use of drug supply in order to maximize both short-term population-wide benefits and long-term epidemiological effects of antiviral therapy. In this study, we developed a mathematical model to assess the impact of various antiviral strategies on curtailing disease, by considering the interplay between three confounding factors: (i) the treatment level of clinical infections; (ii) the emergence and spread of antiviral resistance; and (iii) the size of the stockpile. In the absence of resistance, we have shown that an intensive treatment early on during the outbreak minimizes the overall disease burden regardless of the size of the stockpile. In this case, if *R*_0 _is not too high, containment of the disease may be achieved with sufficiently high level of treatment. This strategy is particularly beneficial when stockpile is limited, since it significantly reduces the spread of disease in the population, and therefore requires fewer courses of antiviral drugs.

Our results suggest a significantly different strategy for antiviral use if resistance were to develop with a transmission fitness comparable to that of the sensitive strain. As indicated by simulations (Figures 3, 4), emergence of drug-resistance has no considerable impact on the use of drugs, and therefore on the depletion of stockpiles, if treatment is maintained at low levels throughout the outbreak, or *R*_0 _is sufficiently high. However, for moderate to high treatment levels, the spread of resistance leads to a more rapid consumption of available stockpiles, and run-out is likely to occur even for low values of *R*_0 _(Figure 4b–c). For comparison purposes, we applied an adaptive antiviral strategy that has been thoroughly evaluated in previous work [19], and observed that delaying aggressive treatment can potentially eliminate the possibility of wide-spread drug resistance, and also minimize the final size of the outbreak. This strategy allows for the initial prevalence of the drug-sensitive strain under low pressure of drugs to deplete a sizable portion of susceptible hosts [18], and therefore prevents the outgrowth of resistance when selection occurs. However, a timely increase in the scale of drug use plays a critical role in the success of this adaptive treatment policy. We demonstrated that, if high treatment levels are implemented too early during the outbreak, a second peak of infections can occur due to run-out with limited stockpile, or as a result of population-wide spread of resistance (Figure 6a–c).

A comparative evaluation of antiviral use indicates that the overall healthcare benefits of an adaptive strategy may be much higher than a constant treatment policy. Assuming *R*_0 _= 2, the adaptive strategy with an initial 0% treatment level (increased to 80% at time t* = 35) requires a stockpile of size 18.5% (relative to *S*_0_), and results in 24% reduction in the total number of clinical infections compared with the constant antiviral treatment at the optimal level 41%, which requires a stockpile of size 17.5%. We observed a similar outcome of the adaptive strategy with 25% initial treatment level (increased to 80% at time t* = 50) and 19% stockpile, leading to 22% reduction in the total number of clinical infections compared with the constant treatment at the optimal level. While the adaptive treatment policy places a demand for slightly larger stockpiles, its increased financial burden must be weighted against the inevitably far greater cost savings that would be obtained through substantial reduction in morbidity and therefore hospitalizations during the pandemic.

For a novel influenza strain with the reproduction number similar to that of the 1918 pandemic [20,29], the findings suggest that in order to reduce the risk of a subsequent wave of infections within an adaptive treatment strategy, pandemic plans should consider stockpiling antiviral drugs with a minimum capacity of 20% (relative to the population size). Given that drugs may also be used for pre-exposure prophylaxis of front-line healthcare workers and emergency responders, and considering that prophylaxis makes a greater contribution to the spread of resistance [25], much larger stockpiles would be required to avoid run-out. It is, however, suggested that allocating different drugs for treatment and prophylaxis may constrain resistance development in the population, and therefore reduce the use of antiviral courses [32].

Our efforts in this study are based on simulating a compartmental epidemic model using parameters estimated in the published literature that involve some degree of uncertainty, particularly with regard to the duration of asymptomatic infection and the effectiveness of antiviral treatment. For the impact of antivirals, we assumed a 60% reduction in absolute infectiousness from the start of treatment, which is consistent with a recent meta-analysis of antiviral effects on secondary attack rates observed in household studies [33]. Since exposed individuals cannot transmit the disease during the short period of latency, we simplified the model to exclude the classes of exposed individuals. Furthermore, treatment of infected individuals is not feasible until after the latent period has elapsed and may be initiated upon diagnosis during symptomatic infection. Although the inclusion of these classes and delay in start of treatment more realistically represents the epidemiology of disease [19], the results are expected to alter quantitatively. While emphasizing the qualitative aspects of the results, we understand that this modelling approach is subjected to several limitations, particularly with regard to heterogeneity in population interactions and stochastic effects at the early stages of an outbreak. There is also much uncertainty about the parameters governing resistance in vivo [14], and how a novel influenza strain would affect different populations with distinctly different mobility patterns [34]. Nonetheless, combined with the previous work on strategic use of drugs for reducing the likelihood of resistance emergence [19], our results suggest that prolonging the effectiveness of antiviral drugs would need to be considered in practical implementation of treatment strategies, especially with the expected delay in availability of a strain-specific vaccine. We should point out that in our model, there is no parameter quantifying the detection of the outbreak. However, in the case of pandemic influenza, the previous threshold used for identification of seasonal outbreaks when ~1 – 5% of the population has been infected seems unrealistically high [35], especially in light of the ongoing surveillance and also recent experience with SARS and other emerging infectious diseases. Furthermore, given the uncertainty of parameter estimation, the timing results of this work should not be interpreted quantitatively, but rather as a general principle for antiviral strategies that delaying the onset of wide-scale treatment can potentially reduce the overall disease burden while preventing large resistant outbreaks.

## Conclusion

Our simulations show that a second wave of infections can occur due to the emergence of highly transmissible resistance or as a result of run-out under the scenario of limited antiviral stockpile. The results demonstrate that conservative treatment levels during the early stages of the outbreak, followed by a timely increase in the scale of drug-use, can minimize the likelihood of both resistance emergence and run-out. The findings suggest that, for a 1918-like influenza virus, pandemic plans should consider stockpiling antiviral drugs for treatment of at least 20% of the population.

## Competing interests

The authors declare that they have no competing interests.

## Authors' contributions

Developed the model: JA, CB, SM. Analyzed the model theoretically: JA.

Conceived and performed the experiments: CB. Designed the study and wrote the paper: SM. All the authors have read and approved the final version of this paper.

## Pre-publication history

The pre-publication history for this paper can be accessed here:

http://www.biomedcentral.com/1471-2334/9/8/prepub

## Supplementary Material

Additional file 1 **Antiviral resistance during pandemic influenza: implications for stockpiling and drug use.** Model structure and its sensitivity analyses with parameter values are provided. Click here for file

## References

[B1] Jennings LC Peiris M Avian influenza H5N1: is it a cause for concern? Intern Med J 2006 36 3 145 147 16503947 10.1111/j.1445-5994.2006.01036.x

[B2] WHO 2008 http://www.who.int/csr/disease/avian_influenza/country/cases_table_2008_06_19/en/index.html

[B3] Ferguson NM Cummings DAT Cauchemez S Fraser C Riley S Meeyai A Iamsirithaworn S Burke DS Strategies for containing an emerging influenza pandemic in Southeast Asia Nature 2005 437 209 214 10.1038/nature04017 16079797

[B4] Ferguson NM Cummings DAT Fraser C Cajka J Cooley P Burke D Strategies for mitigating an influenza pandemic Nature 2006 442 448 452 10.1038/nature04795 16642006 PMC7095311

[B5] Gani R Hughes H Fleming D Griffin T Medlock J Leach S Potential impact of antiviral drug use during influenza pandemic Emerg Infect Dis 2005 11 9 1355 1362 16229762 10.3201/eid1109.041344 PMC3371825

[B6] Longini IM Jr Halloran ME Nizam A Yang Y Containing pandemic influenza with antiviral agents Am J Epidemiol 2004 159 623 633 10.1093/aje/kwh092 15033640

[B7] Longini IM Jr Nizam A Xu S Ungchusak K Hanshaoworakul W Cummings DAT Halloran ME Containing pandemic influenza at the source Science 2005 309 1083 1087 10.1126/science.1115717 16079251

[B8] WHO checklist for influenza pandemic preparedness planning 2005 http://www.who.int/csr/resources/publications/influenza/WHO_CDS_CSR_GIP_2005_4/en/

[B9] Bridges CB Thompson WW Meltzer MI Reeve GR Talamonti WJ Cox NJ Lilac HA Hall H Klimov A Fukuda K Effectiveness and cost-benefit of influenza vaccination of healthy working adults: A randomized controlled trial JAMA 2000 184 1655 1663 10.1001/jama.284.13.1655 11015795

[B10] Democratis J Pareek M Stephenson I Use of neuraminidase inhibitors to combat pandemic influenza J Antimicrob Chemother 2006 58 911 915 10.1093/jac/dkl376 16956904

[B11] Inglesby TV Nuzzo JB O'Toole T Henderson DA Disease mitigation measures in the control of pandemic influenza Biosecur Bioterr 2006 4 1 10 10.1089/bsp.2006.4.1 17238820

[B12] Alexander ME Bowman CS Feng Z Gardam M Moghadas SM Röst G Wu J Yan P Emergence of drug-resistance: implications for antiviral control of pandemic influenza Proc R Soc B 2007 274 1675 1684 17507331 10.1098/rspb.2007.0422 PMC2493585

[B13] de Jong MD Thanh TT Khanh TH Hien VM Smith GJG Chau NV Cam BV Qui PT Ha DQ Guan Y Peiris JSM Hien TT Farrar J Oseltamivir resistance during treatment of influenza A (H5N1) infection N Engl J Med 2005 353 2667 2672 10.1056/NEJMoa054512 16371632

[B14] Handel A Longini IM Jr Antia R Neuraminidase inhibitor resistance in influenza: assessing the danger of its generation and spread PLoS Comput Biol 2007 3 e240 18069885 10.1371/journal.pcbi.0030240 PMC2134965

[B15] Kiso M Mitamura K Sakai-Tagawa Y Shiraishi K Kawakami C Kimura K Hayden FG Sugaya N Kawaoka Y Resistant influenza A viruses in children treated with oseltamivir: descriptive study Lancet 2004 364 759 765 10.1016/S0140-6736(04)16934-1 15337401

[B16] Moscona A Oseltamivir resistance – disabling our influenza defenses N Engl J Med 2005 353 2633 2636 10.1056/NEJMp058291 16371626

[B17] Yen HL Herlocher LM Hoffmann E Matrosovich MN Monto AS Webster RG Govorkova EA Neuraminidase inhibitor-resistant influenza viruses may differ substantially in fitness and transmissibility Antimicrob Agents Chemother 2005 49 4075 4084 16189083 10.1128/AAC.49.10.4075-4084.2005 PMC1251536

[B18] Moghadas SM Management of drug-resistance in the population: influenza as a case study Proc R Soc B 2008 275 1163 1169 10.1098/rspb.2008.0016 18270154 PMC2602698

[B19] Moghadas SM Bowman CS Röst G Wu J Population-wide emergence of antiviral resistance during pandemic influenza PLoS ONE 2008 3 e1839 18350174 10.1371/journal.pone.0001839 PMC2266801

[B20] Viboud C Tam T Fleming D Handel A Miller MA Simonsen L Transmissibility and mortality impact of epidemic and pandemic influenza, with emphasis on the unusually deadly 1951 epidemic Vaccine 2006 24 6701 6707 10.1016/j.vaccine.2006.05.067 16806596

[B21] Arinaminpathy N McLean AR Antiviral treatment for the control of pandemic influenza: some logistical constraints J R Soc Interface 2008 5 545 553 10.1098/rsif.2007.1152 17725972 PMC3226978

[B22] Handel A Regoes RR Antia R The role of compensatory mutations in the emergence of drug resistance PLoS Comput Biol 2006 2 1262 1270 10.1371/journal.pcbi.0020137 PMC1599768 17040124

[B23] Carr J Ives J Kelly L Lambkin R Oxford J Mendel D Tai L Roberts N Influenza virus carrying neuraminidase with reduced sensitivity to oseltamivir carboxylate has altered properties in vitro and is compromised for infectivity and replicative ability in vivo Antiviral Res 2002 54 79 88 10.1016/S0166-3542(01)00215-7 12062393

[B24] Herlocher ML Carr J Ives J Elias S Truscon R Roberts N Monto AS Influenza virus carrying an R292K mutation in the neuraminidase gene is an transmitted in ferrets Antiviral Res 2002 54 99 111 10.1016/S0166-3542(01)00214-5 12062395

[B25] Regoes RR Bonhoeffer S Emergence of drug-resistant influenza virus: population dynamical considerations Science 2006 312 389 391 10.1126/science.1122947 16627735

[B26] Weinstock DM Zuccotti G Adamantane Resistance in Influenza A JAMA 2006 295 934 936 10.1001/jama.295.8.jed60009 16493107

[B27] Lipsitch M Cohen T Murray M Levin BR Antiviral resistance and the control of pandemic influenza PLoS Med 2007 4 e15 17253900 10.1371/journal.pmed.0040015 PMC1779817

[B28] Arino J Brauer F Driessche P van den Watmough J Wu J A final size relation for epidemic models Math Biosci Eng 2007 4 159 175 17658921 10.3934/mbe.2007.4.159

[B29] Mills CE Robins JM Lipsitch M Transmissibility of 1918 pandemic influenza Nature 2004 432 904 906 10.1038/nature03063 15602562 PMC7095078

[B30] Balicer RD Huerta M Davidovitch N Grotto I Cost-benefit of stockpiling drugs for influenza pandemic Emerg Infect Dis 2005 11 1280 1282 16102319 10.3201/eid1108.041156 PMC3320484

[B31] Siddiqui MR Edmunds WJ Cost-effectiveness of antiviral stockpiling and near-patient testing for potential influenza pandemic Emerg Infec Dis 2008 14 267 274 10.3201/eid1402.070478 PMC2600182 18258120

[B32] McCaw JM Wood JG McCaw CT McVernon J Impact of emerging antiviral drug resistance on influenza containment and spread: influence of subclinical infection and strategic use of a stockpile containing one or two drugs PLoS ONE 2008 3 e2362 18523549 10.1371/journal.pone.0002362 PMC2390853

[B33] Halloran ME Hayden FG Yang Y Longini IM Jr Monto AS Antiviral effects on influenza viral transmission and pathogenicity: observations from household-based trials Am J Epidemiol 2007 165 212 221 10.1093/aje/kwj362 17088311

[B34] Débarre F Bonhoeffer S Regoes RR The effect of population structure on the emergence of drug-resistance during pandemic influenza J R Soc Interface 2007 4 893 906 17609176 10.1098/rsif.2007.1126 PMC2394556

[B35] Stilianakis NI Perelson AS Hayden FG Emergence of drug resistance during an influenza epidemic: insights from a mathematical model J Infect Dis 1998 177 863 873 9534957 10.1086/515246

