# Supplementary material for: Antiviral resistance during pandemic influenza: implications for stockpiling and drug use
Source: BMC Infect Dis. 2009 Jan 22;9:8. doi: 10.1186/1471-2334-9-8 (PMC2653495; doi:10.1186/1471-2334-9-8)
Supplement: Additional file 1 — Antiviral resistance during pandemic influenza: implications for stockpiling and drug use. Model structure and its sensitivity analyses with parameter values are provided. [file 1471-2334-9-8-S1.pdf]

# Supplementary Material
# Antiviral resistance during pandemic influenza: implications for stockpiling and drug use

Julien Arino[1,2], Christopher S. Bowman[2,3] and Seyed M. Moghadas[*,2,4,5]

[1]Department of Mathematics, University of Manitoba,Winnipeg, Manitoba, Canada R3T 2N2
[2]Institute for Biodiagnostics, National Research Council Canada, Winnipeg, Manitoba, Canada R3B 1Y6
[3]Department of Electrical and Computer Engineering, The University of Manitoba, Winnipeg, Manitoba, Canada R3T 5V6
[4]Department of Mathematics and Statistics, University of Winnipeg, Winnipeg, Manitoba, Canada R3B 2E9
[5]Department of Statistics, University of Manitoba, Winnipeg, Manitoba, Canada R3T 2N2

Email: Julien Arino - arinoj@cc.umanitoba.ca; Christopher S. Bowman - chris.bowman@nrc-cnrc.gc.ca; Seyed M. Moghadas - seyed.moghadas@nrc-cnrc.gc.ca;

*Corresponding author

## The core model

Since the model is applied to a pandemic scenario with a short duration of the outbreak compared with the average life-time, we ignored the effects of inflow/outflow rates of birth and natural death on the dynamics of disease transmission. The subpopulations considered in the model include:

$S$: susceptible

$A$: asymptomatic infection with sensitive strain

$I_U$: untreated symptomatic infection with sensitive strain

$I_T$: treated symptomatic infection with sensitive strain

$I_{T,r}$: treated symptomatic infection with resistant strain (LTF)

$A_r$: asymptomatic infection with resistant strain (HTF)

$I_{r,U}$: untreated symptomatic infection with resistant strain (HTF)

$I_{r,T}$: treated symptomatic infection with resistant strain (HTF)

$R_U$: removed from sensitive infection without treatment

$R_T$: removed from sensitive infection with treatment

$R_{T,r}$: removed from resistant infection (LTF) with treatment

$R_{r,U}$: removed from resistant infection (HTF) without treatment

$R_{r,T}$: removed from resistant infection (HTF) with treatment

Taking into account the above assumptions, the model can be expressed as the following system of deterministic differential equations:

$$S' = -(f+g)S, \tag{1}$$

$$\text{drug-sensitive infections} \begin{cases} A' = (1-p)fS - \mu_A A, \\ I'_U = (1-q)pfS - (d_U + \mu_U)I_U, \\ I'_T = qpfS - (d_T + \mu_T)I_T - \alpha I_T, \end{cases} \tag{2}$$

$$\text{drug-resistant infections (LTF)} \begin{cases} I'_{T,r} = \alpha I_T - (d_{U,r} + \mu_U)I_{T,r} - \gamma I_{T,r}, \end{cases} \tag{3}$$

$$\text{drug-resistant infections (HTF)} \begin{cases} A'_r = (1-p)gS - \mu_A A_r, \\ I'_{r,U} = (1-q)pgS - (d_{r,U} + \mu_U)I_{r,U}, \\ I'_{r,T} = qpgS + \gamma I_{T,r} - (d_{r,U} + \mu_U)I_{r,T}, \end{cases} \tag{4}$$

where

$$f = \beta(\delta_A A + I_U + \delta_T I_T),$$

$$g = \delta_r \beta(\delta_A A_r + I_{r,U} + I_{r,T}),$$

and the prime ‘ $'$ ’ denotes the derivative of the numbers in compartments with respect to the time; $\beta$ is the baseline transmission rate of the sensitive strain; $p$ is the probability of developing clinical symptoms; $\delta_A$ represents the relative transmissibility of asymptomatic infection; $\delta_T$ is the relative transmissibility of treated individuals infected with the sensitive strain; $\delta_r$ represents the relative transmissibility of the resistant strain (high fitness); $d_U$ and $d_T$ are disease-induced death rates of untreated and treated individuals infected with the sensitive strain, respectively; $d_{U,r}$ and $d_{r,U}$ are disease-induced death rates of individuals infected with mutants of LTF and HTF, respectively; $\mu_A$ is the recovery rate of asymptomatic infection; $\mu_U$ and $\mu_T$ represent recovery rates of untreated and treated infected individuals, respectively; $\alpha$ is the rate at which treated individuals develop drug-resistance (rate of de novo resistant mutation); $\gamma$ is the conversion rate of resistant strains with LTF to HTF; and $q$ is the fraction of infected individuals which receives treatment (treatment level).

The equations for removed subpopulations are given by

$$\begin{aligned}
R'_{U} &= \mu_{A} A + (d_{U} + \mu_{U}) I_{U}, \\
R'_{T} &= (d_{T} + \mu_{T}) I_{T}, \\
R'_{T,r} &= (d_{U,r} + \mu_{U}) I_{T,r}, \\
R'_{r,U} &= \mu_{A} A_r + (d_{r,U} + \mu_{U}) I_{r,U}, \\
R'_{r,T} &= (d_{r,U} + \mu_{U}) I_{r,T},
\end{aligned} \tag{5}$$

where these compartments include both recovered and dead individuals following infection.

While national pandemic plans consider stockpiling antiviral drugs, it is imperative to evaluate the impact of emergence of resistance on antiviral use, particularly for the scenario of limited supply in which run-out is likely to occur. In order to demonstrate the relationship between the level of treatment and drug stockpile, we appended the following equation to the model

$$M(t) = M_0 - \big(I_{T}(t) + I_{T,r}(t) + I_{r,T}(t) + R_{T}(t) + R_{T,r}(t) + R_{r,T}(t)\big),$$

where $M_0$ is the initial size of the stockpile and $M(t)$ represents the number of antiviral courses available at time $t$. We assumed that the supply is only depleted through treatment of indexed cases with a single course of oseltamivir during symptomatic infection, and no additional antiviral courses will be provided during the pandemic.

## Control reproduction number

We applied a previously established technique to calculate $R_c$ [1], and considered infectious classes in the order $X = (A, I_{U}, I_{T}, A_r, I_{r,U}, I_{r,T}, I_{T,r})$ to simplify computations. The system (2)-(4) can be written as $X' = \mathscr{F} - \mathscr{V}$, where

$$\mathscr{F} = \begin{pmatrix} (1-p)fS \\ p(1-q)fS \\ pqfS \\ (1-p)gS \\ p(1-q)gS \\ pqgS \\ 0 \end{pmatrix}$$

and

$$\mathscr{V} = \begin{pmatrix} \mu_{A} A \\ (d_{U} + \mu_{U}) I_{U} \\ (d_{T} + \mu_{T} + \alpha) I_{T} \\ \mu_{A} A_r \\ (d_{r,U} + \mu_{U}) I_{r,U} \\ -\gamma I_{T,r} + (d_{r,U} + \mu_{U}) I_{r,T} \\ -\alpha I_{T} + (d_{U,r} + \mu_{U} + \gamma) I_{T,r} \end{pmatrix}.$$

Taking the Fréchet derivatives $F = D\mathscr{F}$ and $V = D\mathscr{V}$, and evaluating at the disease free equilibrium $(S_0, X = 0)$, we have

$$F = \begin{pmatrix} F_{11} & 0 \\ 0 & F_{22} \end{pmatrix},$$

with

$$F_{11} = \begin{pmatrix} (1-p)\delta_A & (1-p) & (1-p)\delta_T \\ p(1-q)\delta_A & p(1-q) & p(1-q)\delta_T \\ pq\delta_A & pq & pq\delta_T \end{pmatrix} \beta S_0,$$

$$F_{22} = \begin{pmatrix} (1-p)\delta_r\delta_A & (1-p)\delta_r & (1-p)\delta_r & 0 \\ p(1-q)\delta_r\delta_A & p(1-q)\delta_r & p(1-q)\delta_r & 0 \\ pq\delta_r\delta_A & pq\delta_r & pq\delta_r & 0 \\ 0 & 0 & 0 & 0 \end{pmatrix} \beta S_0$$

and

$$V = \begin{pmatrix} V_{11} & 0 \\ V_{21} & V_{22} \end{pmatrix},$$

with

$$V_{11} = \mathsf{diag}(\mu_A, d_U + \mu_U, d_T + \mu_T + \alpha, \mu_A, d_{r,U} + \mu_U),$$

$$V_{21} = \begin{pmatrix} 0 & 0 & 0 & 0 & 0 \\ 0 & 0 & -\alpha & 0 & 0 \end{pmatrix},$$

$$V_{22} = \begin{pmatrix} d_{r,U} + \mu_U & -\gamma \\ 0 & d_{U,r} + \mu_U + \gamma \end{pmatrix}.$$

Then, by evaluating $V^{-1}$, the control reproduction number is defined by the spectral radius of $FV^{-1}$ and given by $R_c = \max\{R_c^s, R_0^r\}$, where $R_c^s$ and $R_0^r$ are expressed in (1) and (2) of the main text, respectively.

### Final size relation: no transmissible resistant strains

Without a transmissible resistant strain ($\gamma = 0$), and assuming that resistant strains are present only with LTF, the model reduces to (1)-(3) with $g \equiv 0$, and the control reproduction number is $R_c^s$. Integrating (1) for the entire course of the outbreak gives

$$\ln\Big(\frac{S_\infty}{S_0}\Big) = -\beta \int_0^\infty \big(\delta_A A + I_U + \delta_T I_T\big) dt, \tag{6}$$

where $S_0$ and $S_\infty$ are respectively the initial and final sizes of the susceptible population. Integrating $(1-p)S' + A'$, $(1-q)pS' + I_U'$, and $qpS' + I_T'$, and substituting into (6), we can express the final size equation as

$$\ln\Big(\frac{S_0}{S_\infty}\Big) = R_c^s\Big(1 - \frac{S_\infty}{S_0}\Big) + \frac{\beta I_U(0)}{d_U + \mu_U}, \tag{7}$$

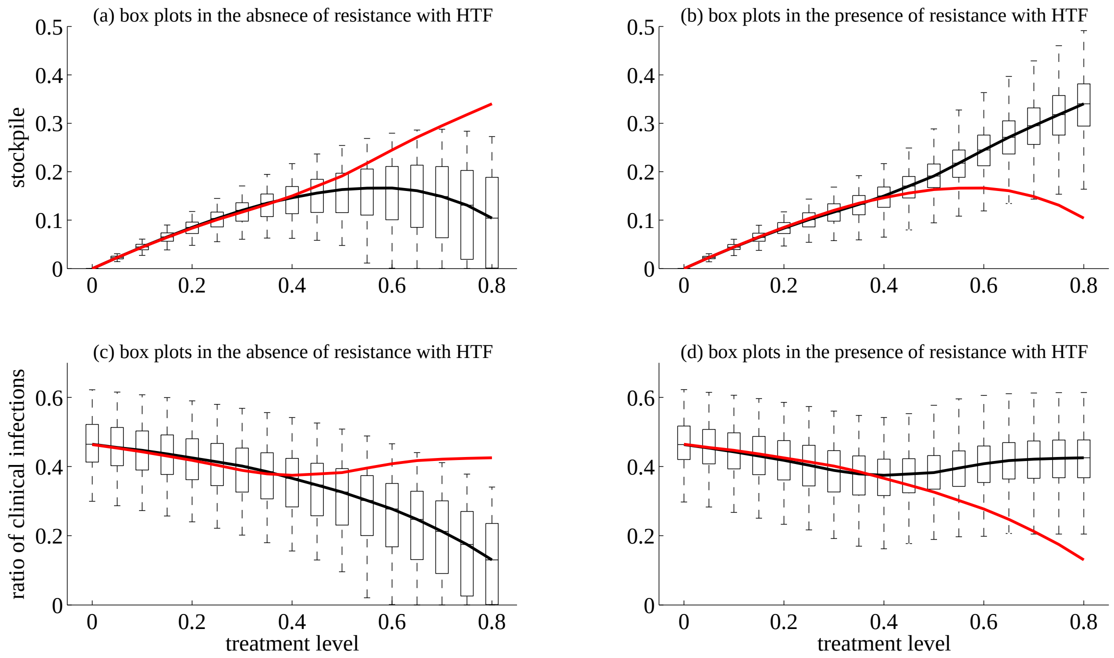


Figure 1: Sensitivity analyses showing box plots for the levels of stockpile and the ratios of the total number of infections in the absence of resistance with HTF (a,c), and presence of resistance with HTF (b,d). The solid black curves pass through the median values, and each box contains 50% of data points between the first and third quartiles of the sampling distribution. The remaining 50% of data points are represented by whiskers. Red curves pass through the median values of the corresponding figures with or without resistance.

where $I_U(0)$ is the initial number of infections at the onset of the outbreak. By integrating equation (3), we can also compute the total number of resistant cases resulted from the treatment of sensitive infections as

$$R^{\infty}_{T,r} \equiv \int_0^{\infty} R'_{T,r}\,dt = \frac{qp\alpha(S_0 - S_{\infty})}{d_T + \mu_T + \alpha}. \tag{8}$$

## Sensitivity analyses

To investigate the effect of parameter changes on the results presented by simulations in the main text using baseline values, we performed sensitivity analyses by considering a sampling approach that allows for the simultaneous variations of several key parameters, including the basic reproduction number $R_0$, the rate of de novo resistant mutations $\alpha$, the rate of conversion between resistant strains $\gamma$, the relative transmissibility of the resistant strain $\delta_r$, and the probability of developing clinical disease $p$. Using the Latin Hypercube Sampling technique [2], we generated samples of size $n = 1000$ in which each parameter is treated as a random variable and assigned a probability function. In this technique, the parameters are

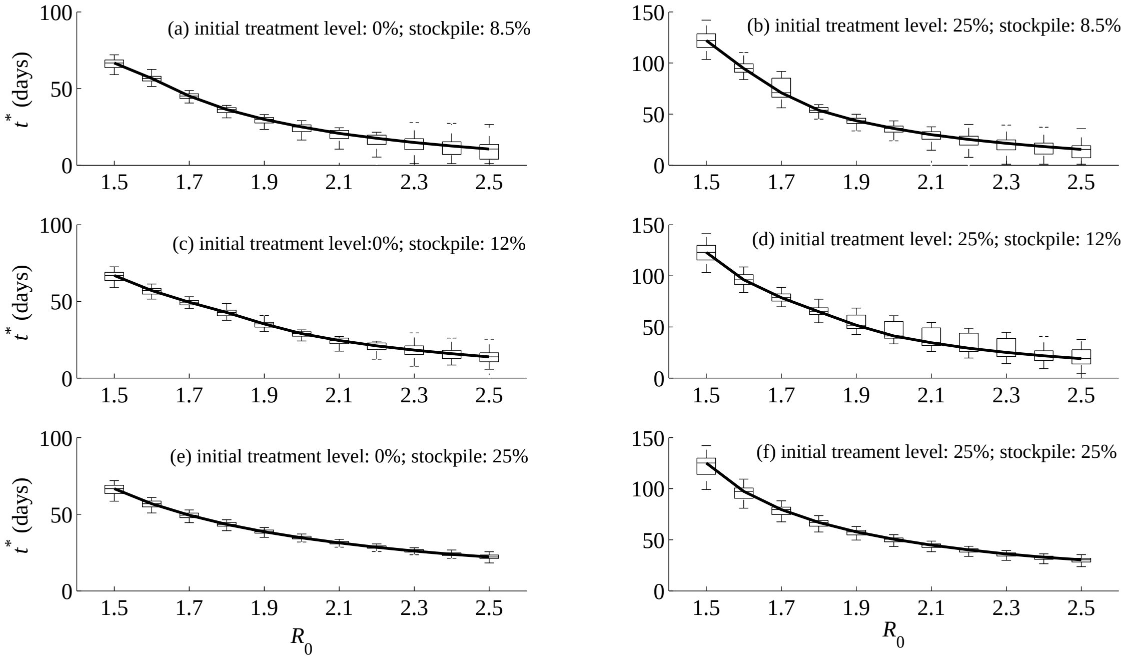


Figure 2: Sensitivity analyses showing box plots for the variations in the optimal transition time $t^*$, corresponding to the minimum total number of infections (including both sensitive and resistant cases), as a function of $R_0$, with other parameters sampled from their respective ranges, as described in the text. The solid curves pass through the median values of data points for $t^*$, and each box contains 50% of data points between the first and third quartiles of the sampling distribution. The remaining 50% of data points are represented by whiskers. Initial treatment levels in adaptive antiviral strategy before transition time $t^*$ are: (a,c,e) 0%; (b,d,f) 25%. The treatment level changes to 80% at time $t^*$, and levels of stockpile are: (a,b) 8.5%; (c,d) 12%; (e,f) 25%.

uniformly distributed and sampled within their respective ranges. The reproduction number was uniformly sampled from the range [1.5, 2.5] (values found in references [3,4]), and the rate of de novo resistant emergence was sampled from the range [0.018, 0.072] (values taken from references [5,6]). The corresponding range for the conversion rate of resistant strains was computed using the constraint that the fraction of treated individuals hosting resistance, which undergoes compensatory mutations and subsequently generates resistant strains with high fitness, lies between 1/5000 and 1/500 [7,8]. Furthermore, we considered a range of [0.5, 0.7] for the probability of developing clinical disease [9,10], and sampled the relative transmissibility of resistant strain with HTF from the range [0.8, 1] [8]. The same ranges of parameter values were also used in a previous study for sensitivity analyses [8].

To evaluate the effect of parameter changes on the required stockpile and the total number of infections, we ran the simulations for different treatment levels (between 0% and 80%) in the presence/absence of

resistance. These simulations, illustrated in Figure 1, correspond to Figure 2 in the main text, and show that a substantially larger stockpile is required when treatment exceeds a certain level in a constant treatment strategy. This is due to the wide spread of highly transmissible resistance under high pressure of antiviral drugs, which in turn leads to a larger number of total infections. We further ran simulations for the optimal time $t^*$ in an adaptive treatment strategy (using a sample of size $n = 100$), to determine the sensitivity of the results on the parameters variation in minimizing the total number of infections. The results of this sensitivity analysis are illustrated in Figure 2, with different sizes of stockpile (8.5%, 12%, 25%), when initial treatment levels (0%, 25%) change to 80% at time $t^*$. Regardless of the level of stockpiles, the results show that aggressive treatment should be implemented with shorter delay after the onset of the outbreak as the basic reproduction number increases. However, the implementation of intensive treatment requires a significantly longer delay for higher initial treatment levels as the reproduction number decreases.

## References


1. van den Driessche P, Watmough J: **Reproduction numbers and subthreshold endemic equilibria for compartmental models of disease transmission**. *Math. Biosc.* 2002, **180**:29–48.
2. McKay M, Conover W, Beckman R: **A comparison of three methods for selecting values of input variables in the analysis of output from a computer code**. *Technometrics* 1979, **21**:239–245.
3. Mills C, Robins J, Lipsitch M: **Transmissibility of 1918 pandemic influenza**. *Nature* 2004, **432**:904–906.
4. Viboud C, Tam T, Fleming D, Handel A, Miller M, Simonsen L: **Transmissibility and mortality impact of epidemic and pandemic influenza, with emphasis on the unusually deadly 1951 epidemic**. *Vaccine* 2006, **24**:6701–6707.
5. Regoes R, Bonhoeffer S: **Emergence of drug-resistant influenza virus: population dynamical considerations**. *Science* 2006, **312**:389–391.
6. Débarre F, Bonhoeffer S, Regoes R: **The effect of population structure on the emergence of drug-resistance during pandemic influenza**. *J. R. Soc. Interface* 2007, **4**:893–906.
7. Lipsitch M, Cohen T, Murray M, Levin B: **Antiviral resistance and the control of pandemic influenza**. *PLoS Med.* 2007, **4**:e15: 0111–0121.
8. Moghadas S, Bowman C, Röst G, Wu J: **Population-wide emergence of antiviral resistance during pandemic influenza**. *PLoS ONE* 2008, **3**:e1839.
9. Ferguson N, Cummings D, Cauchemez S, Fraser C, Riley S, Meeyai A, Iamsirithaworn S, Burke D: **Strategies for containing an emerging influenza pandemic in Southeast Asia**. *Nature* 2005, **437**:209–214.
10. Longini JI, Nizam A, Xu S, Ungchusak K, Hanshaoworakul W, Cummings D, Halloran M: **Containing pandemic influenza at the source**. *Science* 2005, **309**:1083–1087.
